# ICEP: An Instrumented Cycling Ergometer Platform for the Assessment of Advanced FES Strategies

**DOI:** 10.3390/s23073522

**Published:** 2023-03-28

**Authors:** Petar Kajganic, Vance Bergeron, Amine Metani

**Affiliations:** Univ. Lyon, ENS de Lyon, CNRS, Laboratoire de Physique, F-69342 Lyon, France

**Keywords:** functional electrical stimulation (FES), FES cycling, spinal cord injury (SCI), cycling ergometer, electrical stimulation patterns, stimulation strategy assessment

## Abstract

Background: Functional electrical stimulation (FES) cycling has seen an upsurge in interest over the last decade. The present study describes the novel instrumented cycling ergometer platform designed to assess the efficiency of electrical stimulation strategies. The capabilities of the platform are showcased in an example determining the adequate stimulation patterns for reproducing a cycling movement of the paralyzed legs of a spinal cord injury (SCI) subject. Methods: Two procedures have been followed to determine the stimulation patterns: (1) using the EMG recordings of the able-bodied subject; (2) using the recordings of the forces produced by the SCI subject’s stimulated muscles. Results: the stimulation pattern derived from the SCI subject’s force output was found to produce 14% more power than the EMG-derived stimulation pattern. Conclusions: the cycling platform proved useful for determining and assessing stimulation patterns, and it can be used to further investigate advanced stimulation strategies.

## 1. Introduction

Lower-limb cycling devices are among the most globally available human-powered mechanical devices and are used for locomotive, sport or leisure activities. They come in numerous designs (bicycles, tricycles, recumbent bikes, etc.)—the most important variations being the number of riders, their position on the device (i.e., upright or reclined) and the number of wheels, depending on the specific functional needs [1,2].

As a result, cycling is one of the most widely used forms of exercise to increase cardiovascular health and build lower-limb muscle strength. For these reasons, this type of activity is ideal for individuals with lower-limb deficiencies, such as paralysis after a spinal cord injury (SCI), stroke, Parkinson disease or multiple sclerosis [3,4]. When these conditions are present, the muscles may not respond fully to voluntary commands but can be activated using electrical stimulation. When electrical stimulation is used for functional outcomes, such as cycling, it is referred to as functional electrical stimulation (FES). FES uses weak electrical fields to trigger action potentials, which provoke nerve impulses, leading to muscle contractions. These contractions can then be sequentially activated to complete a movement. When used with cycling for individuals with motor disabilities, it provides an excellent tool for rehabilitation or recreational activity [5,6,7].

Although invented in the 1980s [8], over the last decade, FES cycling has seen an upsurge in interest, in large part due to international sporting events such as the Cybathlon [9,10] and Lyon Cyberdays [11]. This is reflected in the increase in the number of articles published on the topic of FES cycling since 2016: 101 articles in the year 2022, which is more than double the number of articles published any year prior to the Lyon Cyberdays and the first Cybathlon in 2016. The increase in FES cyclists, which include individuals with various types of motor disabilities, has created a growing need for assessment tools in order to determine the most efficient electrical stimulation strategies to adapt to the different needs, such as optimizing the stimulation patterns (the timing in which the cyclist’s leg muscles are activated) in order to achieve a smooth and powerful cycling movement. Furthermore, selecting the stimulation parameters, such as electrical pulse shape and charge density, that will maximize the torque generated by the muscle contraction while minimizing the subsequent muscular fatigue will have a major influence over the cycling efficiency and its potential health benefits [12,13,14,15,16,17].

Despite the growing popularity of FES cycling, instrumented cycling devices for the assessment of FES-cycling strategies are not common. The existing devices are mainly designed around commercially available cycling ergometers or recumbent tricycles. Cadence-controlled cycling ergometers that provide motor and crank position data can be adapted for FES cycling, and the torque produced by the cyclist can be derived from the motor current [18]. Another approach is utilizing the recumbent tricycle by replacing the crank set with a crank power meter [17,19]. Hunt et al. [20] have expanded on this by motorizing the recumbent tricycle and integrating the power meter into the crank, allowing the system to be cadence- and output-power-controlled. Alternatively, an isokinetic knee joint torque measurement system with integrated electrical stimulation can be used to assess FES-cycling strategies by mimicking the knee joint motion during cycling movement [21].

The main limitation of the mentioned devices is versatility. The seating position is predefined and requires a transfer which is often difficult for individuals with certain disabilities. In addition, most of the mentioned devices do not record the torque produced by each leg separately. The torque recorded is one-dimensional, and it corresponds to the component which is perpendicular to the crank arm and contributes to the cycling motion. Thus, the only possible optimization method is to maximize that component. Recording three-dimensional torque allows for optimization techniques that would, in addition, try to minimize the power lost in forces exerted onto the pedals that do not contribute to the cycling motion.

In order to optimize FES-cycling movements and protocols, we developed a novel instrumented cycling ergometer platform (ICEP) designed to evaluate the effectiveness of various FES-cycling strategies. The platform is adaptable to different reclined cycling positions and is outfitted with highly sensitive force sensors to directly measure the effects of different FES-cycling parameters. In addition, subjects can also be tested while seated in their wheelchair so a transfer is not required.

The purpose of the present article is to describe and assess the ICEP. Furthermore, the capabilities of the ICEP are demonstrated through the example of determining and optimizing stimulation patterns. The platform described in this work can be easily reproduced and will hopefully motivate future in-depth specific studies to further progress the number of applications and designs of new FES-cycling devices.

## 2. Materials and Methods

### 2.1. Description of the Equipment

**Figure 1:**
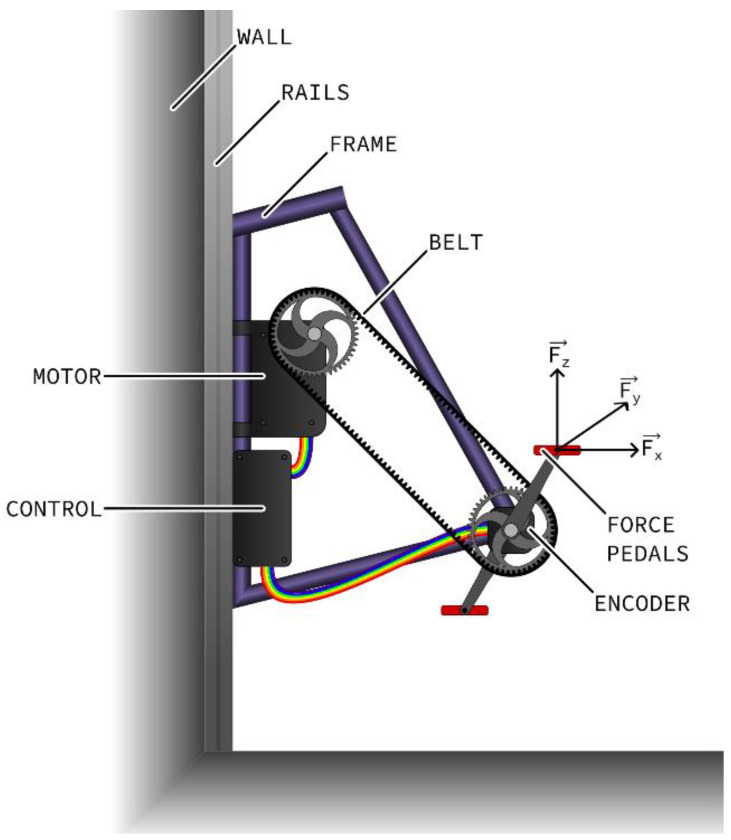
Sketch of the wall-mounted instrumented cycling ergometer platform.

The platform consists of a modified bicycle frame fitted with pedal force/torque sensors, a crank encoder and a motor (Figure 1). The frame is resting on two wall-mounted rails, allowing the ergometer to slide up and down, changing the height of the crank in order to make the system suitable for hand or foot pedaling in a seated or standing position. It is additionally stiffened with tension wires to increase sturdiness, hence limiting the shaking and deformation of the frame caused by cycling.

The actuator system, consisting of a brushless motor (EC60 Flat, Maxon Motor AG, Switzerland) associated with a planetary 53:1 ratio gear-head (GP52C, Maxon, Switzerland), can produce a maximum torque of 228 Nm. It is connected to the crankset through a pulley and belt system (A9-H075, A8-H, Michaud-Chailly, Saint-Priest, France). The motor rotational speed is controlled by a servo controller, and the power is supplied through a shunt regulator (ESCON 70/10, DSR 70/30, Maxon, Sachseln, Switzerland). It is set up to assist the cyclist with producing the cycling movement while maintaining a constant cycling cadence, which can be set from within a custom-made LabVIEW (National Instruments, Austin, TX, USA) graphical interface, with values ranging from 1 rpm to 100 rpm.

Each pedal independently measures all three components of the force and torque applied to their surface, as well as the inclination angle of the pedal in reference to the crank arm (ICS-RM, Sensix, Poitiers, France). Force values up to 2.8 kN can be measured while the inclination angle is measured with a resolution of 0.072°. A magnetic ring encoder (LM13, RLS, Ljubljana, Slovenia) placed around the spindle of the crankset acquires the crank angle with a resolution of 0.06°. Data are acquired at a 1 kHz sampling rate and displayed in real time, as well as recorded in a text file for later analysis.

**Figure 2:**
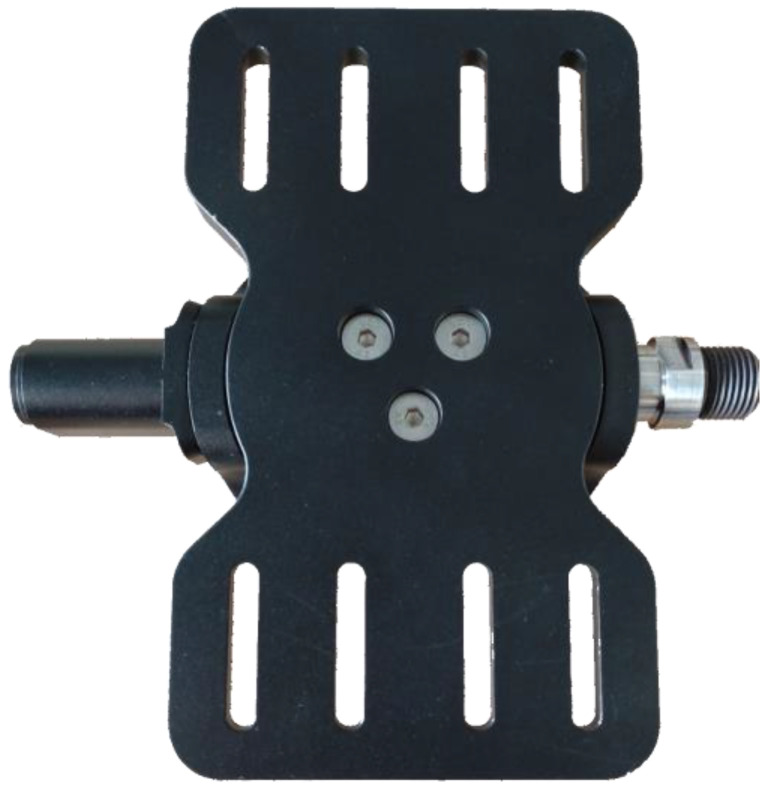
A custom connecting plate attached to the force/torque measuring pedal.

**Figure 3:**
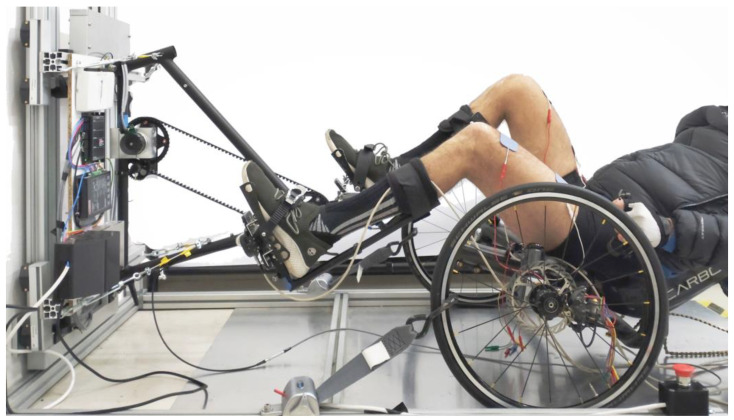
Photo of an SCI subject using the platform from the Carbontrike.

Leg orthoses (Hase Bikes, Waltrop, Germany) were modified to attach the legs to the force-measuring pedals. To ensure that there are zero degrees of movement between the leg orthosis and the pedal, custom connecting metal plates were manufactured (Figure 2) and attached to the pedals, providing a grooved surface to which the orthosis can be attached. The grooves in the metal plates allow for the adjustment of the position of the orthosis, and therefore the foot, in reference to the pedal. The platform was designed to be used from a wheelchair; therefore, it does not have a built-in seat. Cycling from their wheelchairs allows the pilots to avoid transfer and provides more comfort in general. The platform can also be used while seated on a recumbent tricycle, from which the boom (the part of the trike carrying the crankset that slides in and out of the frame to adjust for leg length) has been removed in order to reproduce accurate cycling positions (Figure 3). We used the Carbontrike (Carbontrikes, Bandhagen, Sweden), a custom-made carbon recumbent trike used by the ENS de Lyon team for the 2016 and 2020 editions of Cybathlon’s FES-cycling races. The trike or the wheelchair is secured to the ergometer with a retractor system attached to the ergometer frame and hooked to the wheels (Q’straint, Oakland Park, FL, USA).

A current-controlled electrical stimulator (MotiMove, 3F—Fit Fabricando Faber, Belgrade, Serbia) is synchronized with the force measuring pedals through the custom-made LabVIEW graphical interface. This 8-channel stimulator produces asymmetrical biphasic pulses with exponential compensation [22]. It can be controlled through a dedicated communication protocol, similar to Hasomed Rehastim’s Science Mode, that allows for the following parameters to be preset or changed in real-time for each channel: stimulation mode (singlet or doublet), pulse amplitude (0–170 mA), pulse width (0–1000 µs), frequency (0–100 Hz) and inter-pulse interval in the doublet stimulation mode (2.7–10 ms).

A data acquisition card (PCI-6221, National Instruments, Austin, TX, USA) completes the platform and collects the data from the crank encoder, allowing for the synchronization of the stimulation with the pedaling cadence, as well as triggering the recording of the force measuring pedals, and setting up the motor cadence and stimulation parameters. Both the motor and the stimulator have an emergency stop button that disconnects them from the power supply.

The force-measuring pedals are factory-calibrated by the manufacturer, while the crank angle and pedal inclination encoders are calibrated before starting each new trial. A crank angle of 0° is achieved when the left crank arm is in the forward position and parallel to the ground. Motor rotational speed control was verified for cycling cadences ranging from 10 rpm to 50 rpm in steps of 10 rpm.

### 2.2. Determining Stimulation Patterns

In FES cycling, a stimulation pattern is a set of crank angle intervals defined for when each muscle group should to be stimulated, in order to reproduce a functional cycling movement. Determining these suitable stimulation intervals can be a tedious process as it will vary according to the pilot’s physical condition, morphology, seating position and the number of muscle groups used; moreover, it has to be optimized in relation to the type of exercise (endurance, interval, sprint, etc.) in order to achieve the best compromise between power and fatigue. As a result, determining and optimizing stimulation patterns served as an appropriate application for demonstrating the capabilities of the cycling platform.

In the following parts, we will describe two different methods for determining FES-cycling stimulation patterns: from EMG measurements on an able-bodied subject and from direct measurements of the forces produced by each SCI subject’s muscle group during a crank revolution.

#### 2.2.1. EMG Recording during Volitional Cycling

A straightforward procedure for reproducing an FES-induced cycling movement is to measure the activation timings of an able-bodied subject’s muscle groups during cycling, through EMG recordings, then to apply these timings to the stimulation of the muscle groups of a paralyzed subject in order to try producing a similar motion [23,24].

One able-bodied adult male (26 years old) subject was chosen on the account of him having a similar height (185 cm) and leg dimensions (50 cm calf and 50 cm thigh) to the SCI subject participating in the second part of the study. Similar leg dimensions are important for achieving the same cycling position, with the same basin versus pedalboard geometrical configuration.

EMG activities of five main muscle groups used in cycling (rectus femoris, vastus lateralis, vastus medialis, biceps femoris and semitendinosus) were acquired on the able-bodied subject, using a 6-channel EMG recorder (FreeEMG 100RT, BTS Bioengineering, Garbagnate Milanese, Italy). The electrodes were placed on shaved and cleaned skin, between 1.5 and 2 cm apart, parallel to the muscle fibers, in accordance with the surface electromyography for the non-invasive assessment of muscles (SENIAM) project guidelines [25].

The recordings were performed on the subject’s dominant leg, during constant-cadence motor-assisted cycling. The subject cycled seated in the Carbontrike, legs attached to the force-measuring pedals using leg orthoses, at a cadence of 50 rpm.

**Figure 4:**
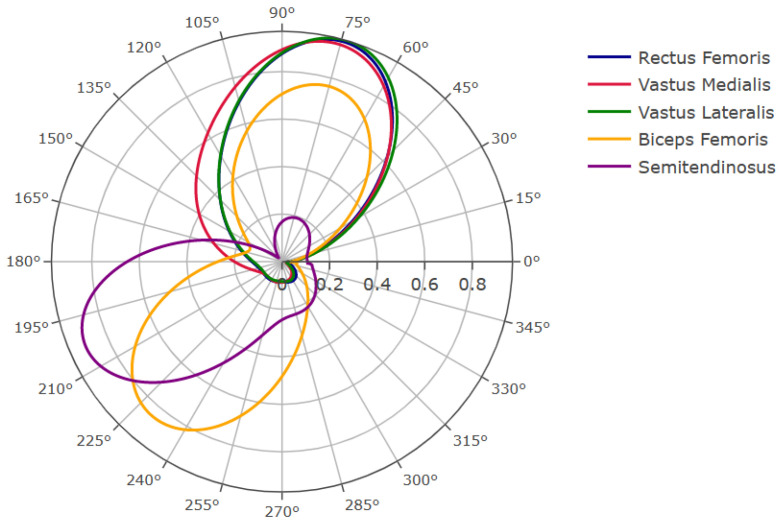
EMG recordings of the five main muscle groups used by the able-bodied subject during cycling (arbitrary normalized unit).

The EMG recordings were processed and analyzed in Matlab (Mathworks, Natick, MA, USA). The raw signals were filtered with a [10 Hz, 30 Hz] 4th order band-pass Butterworth filter. The processed EMG signals were normalized for each revolution and subsequently averaged. The resulting signals were plotted against the crank angles on a 360° plot (Figure 4). They represent the mean EMG profile from where the muscle activation pattern will be determined.

#### 2.2.2. Recording Force Profiles of the Muscle during FES Cycling

An obvious limitation of using an able-bodied cyclist’s EMG recordings to set up a paralyzed pilot’s FES-cycling pattern is that FES only allows for the stimulation of a limited number of muscles that are close to the skin’s surface, whereas volitional cycling also engages deep muscles, such as the iliopsoas muscle, that cannot be reached using non-invasive electrical stimulation. Therefore, the optimal activation timings of a limited number of surface muscles, in order to achieve an effective cycling movement, might substantially differ from the activation timings measured on an able-bodied subject, who would use those surface muscles in combination with additional muscles that remain out of the reach of FES.

**Figure 5:**
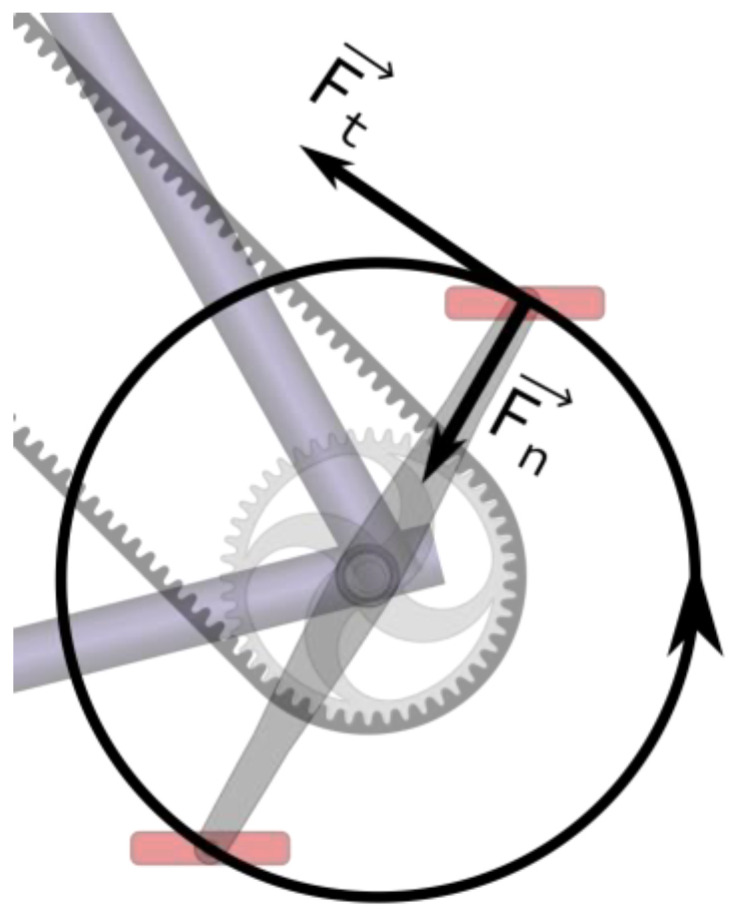
Tangential and normal forces F_t_ & F_n_.

In order to take this limitation into account, a straightforward procedure is to record the tangential force (i.e., the force tangential to the cycling motion, by opposition to the normal force that is parallel to the crank and produces no motion, as illustrated in Figure 5) exerted by a pilot’s foot on each pedal, while continuously stimulating one muscular group during at least one revolution of a motor-assisted cycling motion. In order to only take into account the force produced by the muscle contraction, we need to deduce the forces produced by the feet on the pedals when no muscle is stimulated and the pedaling motion only occurs through motor assistance. We refer to this as passive cycling, in opposition to active cycling when at least one muscle group is stimulated.

**Figure 6:**
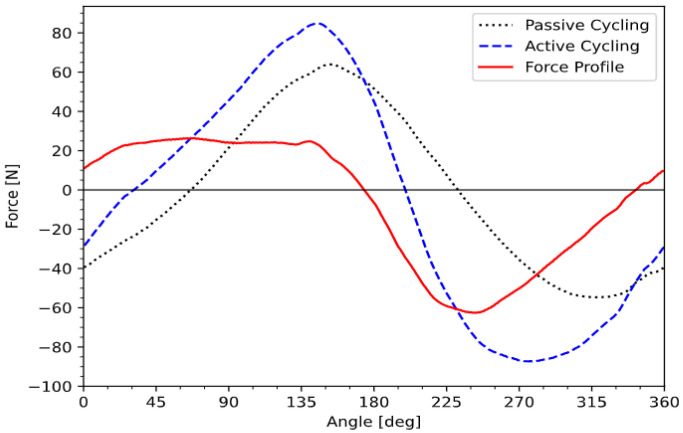
Passive cycling, active cycling and muscle force profile of the right quadricep of the SCI subject.

Other contributing forces, such as the weight of the legs and inertial forces, are similar if passive and active cycling are conducted consecutively in similar conditions (i.e., without changing the seating position or the cadence). By comparing the forces produced during active (stimulation-induced) and passive (motor-assisted) cycling over a complete revolution, we can evaluate the individual contributions of each single muscle group. The difference between active and passive cycling over one revolution (or several averaged revolutions) will be called the muscle’s force profile (MFP) (Figure 6) and used to determine a stimulation pattern.

A positive value of the MFP indicates that the stimulated muscle contributes to cycling in that specific angular range; while a negative value of the force profile indicates an angular range where the stimulated muscle is hindering the cycling motion.

#### 2.2.3. Deriving Stimulation Patterns from EMG and MFP

To derive a stimulation pattern from the EMG recording from Figure 4, an EMG activation interval was defined as the crank angular range during which the mean EMG activation profile was greater than a 25% threshold in comparison to its maximal peak [15]. The activation interval of the quadriceps was then defined as the union of the activation intervals of the rectus femoris, vastus lateralis and vastus medialis. Analogously, the activation interval of the hamstring was defined as the union of the activation intervals of the biceps femoris and semitendinosus. This pattern will thereafter be referred to as the EMG pattern.

Based on Figure 6, if the force profile is positive, the muscle is contributing to the cycling motion; if the force profile is negative, the muscle is resisting the cycling motion. Therefore, the only range of positions suitable for electrical stimulation is where the force profile is positive. In order to derive a stimulation pattern, several procedures could be considered. One could, for instance, detect the maximal force and then set the start angle at the first zero transition before the maximum and the stop angle at the first zero transition after the maximum [14]. Such a range would likely maximize the power output, but also the muscular fatigue. From there, an arbitrary threshold could be defined as a percentage of maximum force, in order to limit the range of stimulation where the produced force is above a certain value. This would allow a compromise between power output and muscle fatigue, hence optimizing the pattern for a less intense but longer exercise.

**Figure 7:**
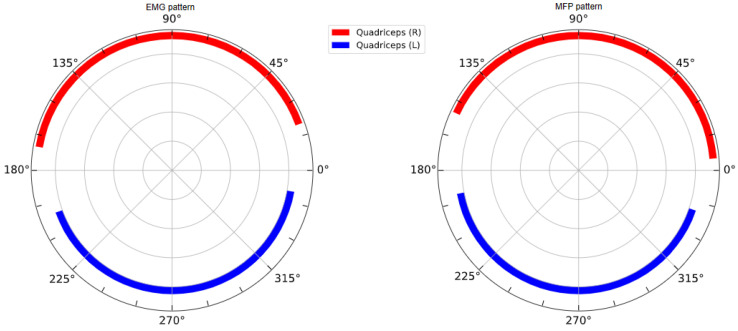
Stimulation patterns for left and right quadriceps derived from EMG recordings on the able-bodied subject (**left**) and from MFP measurement on the SCI subject (**right**).

In order to allow for a fair comparison of the measured power output with the EMG pattern, start and stop angles were chosen so as to have the same angular length as the EMG pattern (and therefore generate the same amount of muscle fatigue) and to maximize the area below the force profile. The resulting pattern will hereafter be referred to as the MFP pattern. Both EMG and MFP patterns are displayed in Figure 7.

#### 2.2.4. Delay Compensation

Regardless of the method chosen to determine the stimulation pattern, various delays must be considered. The delays are a product of the stimulator (1), the measuring equipment (2) and the muscles themselves (3). Estimating or measuring them will allow for compensating for them.

1. The stimulator delay (D_S_) is the internal stimulator delay and represents the time from the moment when the stimulator receives the command to generate a pulse, to the moment the generated pulse arrives to the electrode placed on the skin.
2. The delay caused by the measuring equipment is a product of the acquisition and processing of the crank angle data. It amounts to 1 ms, which, when cycling at 50 rpm, is equivalent to 0.3° of the crank angle. We will thus consider it insignificant.
3. Muscle activation delay, also called electromechanical delay (EMD), is defined as the time it takes for the muscle to develop tension once the stimulation pulse reaches the motor nerve. Muscle delay is usually derived from literature, but it can also be measured.

When recording passive and active profiles in order to measure the MFP, the muscle groups of interest are continuously stimulated. Thus, they are not affected by either of these delays. The delays should be compensated for only when the muscle stimulation follows a specific intermittent pattern.

To ensure that the muscles are activated at the correct position, the angular ranges of the stimulation patterns have to be shifted. To compensate for the stimulator delay, both start and stop angles must be shifted by the same amount. To compensate for the EMD, only the start angle has to be shifted. The required angular shifts depend on the cycling cadence and can be calculated using the following equations:

(1) $$∆\theta_{start}=\frac{\left(D_{s}+EMD\right)\times 360}{60}\omega$$

(2) $$\Delta \theta_{stop}=\frac{D_{s}\times 360}{60}\omega$$

where ∆θ represents the angular shift [°], D_S_ is the stimulator delay [s], EMD is the muscle activation delay [s] and ω is the cycling cadence [rpm].

**Figure 8:**
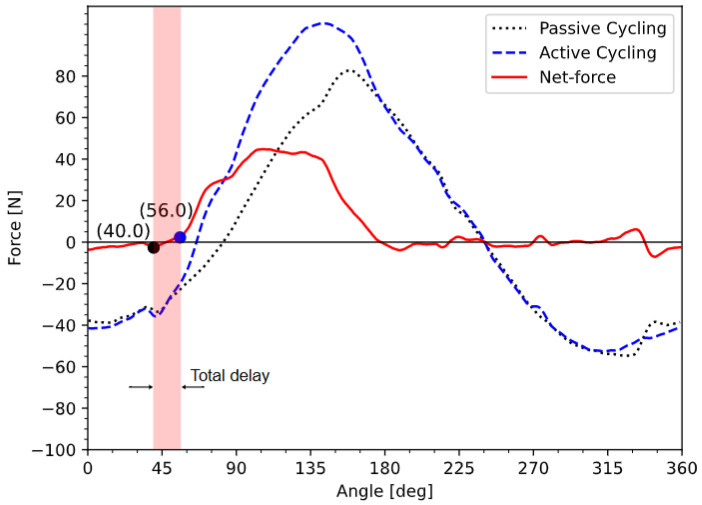
Direct measurement of the angular shift at 50 rpm for the SCI subject.

Angular shifts, and therefore total delay, can also be directly measured by comparing the angle where the stimulation of the muscle begins and the angle where the muscle actually produces a noticeable change in force exerted on the pedals. In Figure 8, the SCI subject’s quadriceps were stimulated at an arbitrary angle, within the first half of the angular range where the MFP is positive, at a cadence of 50 rpm. The “net-force” is the curve obtained by subtracting passive forces from active forces when the muscle group of interest is stimulated only within the angular range defined by the chosen pattern. It represents the effect of the stimulated muscle group on the cycling motion and can be used to measure the total delay and also to calculate the power produced by that muscle group.

#### 2.2.5. Experimental Protocol

One adult male (46 years old) with a motor-complete SCI (lesion level C7-C8, American Spinal Injury Association (ASIA) Impairment Scale (AIS) B) participated in the present case study. The injury occurred 11 years prior to the study. The subject is 184 cm tall, with 50 cm calf and 50 cm thigh lengths. The subject is experienced with FES cycling as he competed in the 2016 and 2020 editions of Cybathlon’s FES-cycling races. He was asked to refrain from exercising at least for 24 h before the experiment as he was involved in the FES-cycling training program at the time of the study.

The medical device used (Motimove 8) was operated strictly following its intended use. As such, the present study does not require the approval of an ethical committee according to French regulations. All procedures followed the usual practices and guidelines regarding rehabilitation of motor function in adult SCI patients, and the subjects gave their written informed consent for study participation.

The experiment was conducted in one session. The session began with a two-minute passive cycling warm-up where the cycling cadence was gradually increased from 10 to 50 rpm. It was followed by four electro-stimulated phases respectively dedicated to:

1. determining the force profiles for each muscle of interest;
2. measuring the total delay;
3. cycling using the EMG pattern;
4. cycling with the MFP pattern.

Each of these four phases began with 5 to 10 passive cycles, followed by 2 active cycles and ended with a 5 min period of rest.

The subject was seated on the Carbontrike in a comfortable position with the legs secured to the ergometer. The trike was placed at the same distance from the pedals as for the EMG test with the able-bodied subject. Using one stimulation channel per leg, pulses were delivered through 9 × 5 cm electrodes (Dura-Stick Premium, Chattanooga, UK) located over the motor points of the quadriceps muscle groups. As the subject has issues with stimulating hamstring muscles, this muscle group was not used. The stimulation frequency was set to 40 Hz, the pulse width to 350 µs and the pulse amplitude to 70 mA. Stimulation parameters were chosen based on the prior experience with FES cycling of the subject, who usually trains 2 to 3 times a week, one-hour sessions on either RT300 (Restorative Therapies, Nottingham, MD, USA) or Motomed (Reck, Betzenweiler, Germany).

After the initial warm-up, the measuring began with obtaining the force profiles. As a compromise between the number of samples acquired and the muscle fatigue produced by the stimulation, the cadence was set to 30 rpm during the first phase. Throughout the other phases of the protocol, the cadence was set to 50 rpm. Following the rest period, the second phase of the protocol was carried out. During the break, recorded data were processed, and the MFP was analyzed using a custom-made Python script. The angular shift was measured to compensate for the delay, and the MFP pattern was set up. During the third and the fourth phases of the protocol, EMG and MFP patterns were respectively used.

The net-forces were calculated from the recorded cycling data. Angular velocity together with the net-forces were used to calculate the mean power produced during one cycle by muscles stimulated with the EMG pattern (P_EMG_) and with the MFP pattern (P_MFP_).

## 3. Results

**Figure 9:**
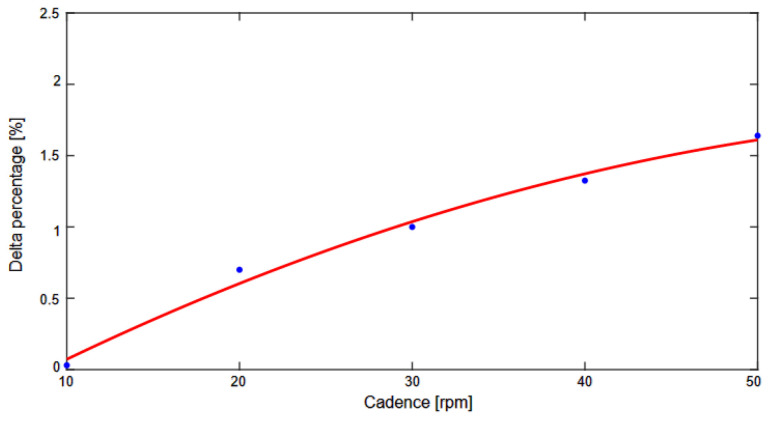
Difference between the target and measured cadence in percentage.

The results of the motor cadence control test are presented in Table 1. The error of the cadence control system increased with the cadence, as shown in Figure 9.

The angular shift was measured to be 16° (Figure 8), and it was applied to the start angle of the EMG and MFP patterns from Figure 7. The angle values for the delay-compensated stimulation patterns are shown in Table 2.

**Figure 10:**
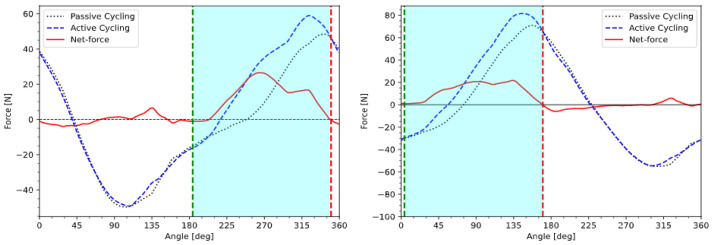
Passive, active and the net-force produced by the left and right quadriceps of the SCI subject stimulated by the EMG pattern. Green and red lines represent the start and stop angles of the delay-compensated EMG pattern, respectively.

**Figure 11:**
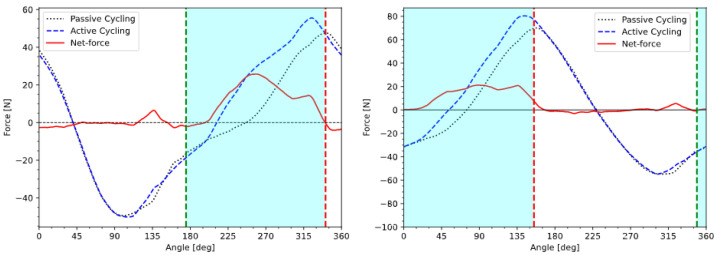
Passive, active and the net-force produced by the left and right quadriceps of the SCI subject stimulated by the MFP pattern. Green and red lines represent the start and stop angles of the delay-compensated MFP pattern, respectively.

The calculated net-forces produced by the quadricep muscles stimulated with the EMG pattern are shown in Figure 10, and the net-forces produced by the quadricep muscles stimulated with the MFP pattern are shown in Figure 11. The mean power produced by the MFP pattern (P_MFP_ = 10.1 W) was 14% greater than the mean power produced by the EMG pattern (P_EMG_ = 8.8 W).

## 4. Discussion

We built a wall-mounted instrumented cycling ergometer, designed to facilitate the assessment of advanced FES-cycling strategies. The motor cadence control accuracy was measured. As an example of the platform’s capabilities, we demonstrated how to determine delay-compensated FES-cycling patterns following two different methods and how to compare their efficiencies.

In order to be able to make a fair comparison between EMG and MFP patterns, we determined the latter so as to generate the same amount of muscular fatigue as the first. This means that after determining the EMG pattern, the MFP pattern was determined by giving it the same angular range while maximizing the total amount of force produced. However, since the angular stimulation range of the EMG pattern was very wide (i.e., 166° which is almost half a revolution), there was little room for variation, and as a consequence, the variation of the power produced when shifting the pattern was limited. Despite being ceiled, the increase in power was still substantial, which is consistent with the results of the previous study [12]. Although encouraging, the patterns need to be further assessed with more subjects in order to achieve statistical relevance. In addition, both patterns should be assessed during longer cycling sessions, specifically to verify the assumption that both patterns induce the same amount of muscle fatigue.

Regardless of the EMG pattern, in order to determine how to optimize the MFP pattern, we need to understand for what type of exercise this pattern is intended. It can be optimized for maximum power production, in which case it would also induce an early onset and rise of fatigue. In that case, the start angle would be set at the first zero transition before the maximum, and the stop angle at the first zero transition after the maximum, which we would call the “full-range” MFP pattern.

However, if the pilot needs to exercise for a substantially longer period of time, a compromise is needed between force and fatigue. In that case, using the full-range MFP pattern is most likely sub-optimal. A threshold would need to be defined as a percentage of the maximum force to be exceeded in order to trigger the stimulation. Optimizing the value of that threshold and quantifying the efficiency of the resulting patterns is typically the type of investigation that this platform facilitates.

When measuring the angular shift with the fresh SCI subject, we found a 16° value which, at the cadence of 50 rpm, represents a total delay of 53.3 ms. Considering that the stimulator delay is constant (3.2 ms), we can deduce that the EMD equals 50.1 ms, which is in concordance with [26]. However, during the course of the experiment, we observed that the angular shift was rising, up to 24° (80 ms). This augmentation correlates with the increase in fatigue of the stimulated muscles. This observation is consistent with the findings of isometric and isokinetic studies measuring the relationship between the EMD and muscle fatigue [26,27,28]. However, other factors, such as the amount of stretch of the muscle (thus the leg position) when the stimulation hits, might also contribute to the increase in the EMD. The relationship between the induced muscle fatigue and the EMD during longer FES-cycling sessions could be investigated using the platform.

One limitation of the present study is that only the quadricep muscles were used for the SCI subject. The latter is a trained FES cyclist that has been using only quadricep muscles and has never trained his gluteal or hamstring muscles. As a consequence, we were not able to get repeatable measurements from these muscles, probably because they were too weak, and possibly spastic. They would need to be progressively trained with TENS prior to FES.

The process of determining the stimulation patterns for additional muscle groups remains identical. However, combining the effects of multiple muscle groups might also introduce uncontrolled muscle synergies [29], which should be investigated in further experiments, along with including more subjects for statistical relevance.

FES strategies that are currently being investigated using the platform include variable-frequency trains and spatially distributed sequential stimulation (SDSS). In order to extend its capabilities, the platform is being continuously improved. For instance, although it was originally designed for FES cycling, the addition of a new motor position control system will allow for its use in isometric studies. A more powerful motor unit could also permit volitional cycling with able-bodied subjects producing higher torques.

## 5. Conclusions

An instrumented cycling ergometer platform for the assessment of advanced FES-cycling strategies was designed and presented in this work. The capabilities of the platform were demonstrated by determining stimulation patterns for the stimulation of paralyzed quadricep muscles. The description of the platform provided in the present article will hopefully enable other research groups to replicate and improve upon this work and, thus, further contribute to the advancement of the assessment of novel stimulation strategies.

## Figures and Tables

**Table 1:** Measured and target cadences in the motor cadence control test.

Target Cadence [rpm]	Measured Cadence [rpm]	Delta Percentage [%]
10	10.003	0.03
20	19.86	0.7
30	29.70	1
40	39.47	1.34
50	49.18	1.66

**Table 2:** Start and stop angles of delay-compensated stimulation patterns for left and right quadriceps, with the power produced by the SCI subject using each of these patterns.

	Left Quadriceps		Right Quadriceps		
	Start Angle [°]	Stop Angle [°]	Start Angle [°]	Stop Angle [°]	Power [W]
**EMG pattern**	184	350	4	170	8.8
**MFP pattern**	175	341	349	155	10.1

## Data Availability

The data and materials presented in this study are available on request from the corresponding author. The data are not publicly available due to protection of privacy.
